# Noni Juice Improves Serum Lipid Profiles and Other Risk Markers in Cigarette Smokers

**DOI:** 10.1100/2012/594657

**Published:** 2012-10-11

**Authors:** Mian-Ying Wang, Lin Peng, Vicki Weidenbacher-Hoper, Shixin Deng, Gary Anderson, Brett J. West

**Affiliations:** ^1^Department of Pathology, University of Illinois College of Medicine at Rockford, 1601 Parkview Avenue, Rockford, IL 61107, USA; ^2^Department of Family and Community Medicine, University of Illinois College of Medicine at Rockford, 1601 Parkview Avenue, Rockford, IL 61107, USA; ^3^Department of Research and Development, Morinda Inc., 737 East 1180 South, American Fork, UT 84003, USA

## Abstract

Cigarette smoke-induced oxidative stress leads to dyslipidemia and systemic inflammation. *Morinda citrifolia* (noni) fruit juice has been found previously to have a significant antioxidant activity. One hundred thirty-two adult heavy smokers completed a randomized, double blind, placebo-controlled clinical trial designed to investigate the effect of noni juice on serum cholesterol, triglyceride, low density lipoprotein cholesterol (LDL), high density lipoprotein cholesterol (HDL), high-sensitivity C-reactive protein (hs-CRP), and homocysteine. Volunteers drank noni juice or a fruit juice placebo daily for one month. Drinking 29.5 mL to 188 mL of noni juice per day significantly reduced cholesterol levels, triglycerides, and hs-CRP. Decreases in LDL and homocysteine, as well increases in HDL, were also observed among noni juice drinkers. The placebo, which was devoid of iridoid glycosides, did not significantly influence blood lipid profiles or hs-CRP. Noni juice was able to mitigate cigarette smoke-induced dyslipidemia, an activity associated with the presence of iridoids.

## 1. Introduction

Cigarette smokers continuously inhale thousands of carcinogens and free radicals. It is estimated that about 10^17^ oxidant molecules are present in each puff of cigarette smoke [1]. Free radicals are known to cause oxidative damage by increasing polymorphonuclear leukocytes and by inducing lipid peroxidation [2, 3]. Additionally, several markers of oxidative stress are elevated in cigarette smokers. These include increased release of reactive oxygen species from phagocytes, such as superoxide from peripheral blood neutrophils, oxidized low density lipoprotein cholesterol (LDL), increased lipid hydroperoxides and malondialdehyde, and decreased plasma antioxidant capacity [4–11].

Among the many adverse health effects from cigarette smoke are dyslipidemia and systemic inflammation. The fact that cigarette smoke increases total cholesterol and triglycerides, as well as decreases high density lipoprotein cholesterol (HDL), has long been established. A meta-analysis of 54 studies revealed that smokers have about 3% higher serum cholesterol and 9% greater serum triglyceride concentrations than nonsmokers. Smokers were also found to have 5.7% lower HDL than nonsmokers [12]. The increases in total cholesterol and triglycerides, with the corresponding decreases in HDL, were found to be dose-dependent when the data were analyzed by smoking frequency. Population-based studies have also revealed that markers of systemic inflammation, such as C-reactive protein (CRP), are also elevated in smokers as well as those exposed to second-hand smoke [13, 14]. Smoking-related elevations in CRP are also accompanied with a rise in serum homocysteine levels [15].

While the molecular mechanisms of tobacco smoke toxicity are still not fully understood, free radical-mediated oxidative stress is believed to play a central role [16, 17]. Oxidative stress, as measured by serum malondialdehyde concentration, is positively correlated to elevated triglyceride and cholesterol levels in smokers [18]. Not only does cigarette smoke increase oxidative stress by increasing free radicals but also by weakening of antioxidant defenses, such as decreasing paraoxonase enzyme activity [19]. These conditions lead to the damage of mitochondria [20], and cigarette smoke may even induce liver injury via lipid peroxidation and corresponding inflammation [21, 22]. Such alterations are likely to lead to imbalances in lipid metabolism.

Fruits and vegetables are major sources of dietary antioxidants. Epidemiological studies indicate that fruits and vegetables may reduce free radical-induced oxidative damage and lipid peroxidation in cigarette smokers [23]. *Morinda citrifolia* (noni) is an evergreen small tree that grows in many tropical regions of the world. Noni fruit has a significant history of use as both food and medicine among Pacific Islanders and in Southeast Asia [24, 25]. Various potential health benefits of noni fruit have been reported [26], including immunomodulation [27, 28] and antioxidant activities *in vitro* and *in vivo* [29–31]. Noni juice has been found to exert an antioxidant effect in human athletes, resulting in increased endurance [32]. Noni juice also lowered plasma concentrations of superoxide anion radicals and lipid hydroperoxides in heavy smokers [33]. Given its demonstrated antioxidant activity, noni juice may also reduce some of the deleterious effects of cigarette smoke. As such, the current study was designed to investigate the influence of noni juice on serum cholesterol, triglyceride, LDL, HDL, high-sensitivity C-reactive protein (hs-CRP), and homocysteine levels in current heavy smokers.

## 2. Material and Methods

### 2.1. Study Ethics

The research protocol of this trial was approved by the Institutional Review Board Committee of the University of Illinois College of Medicine at Rockford. Written informed consent was obtained from all study participants. The trial was conducted in accordance with the Declaration of Helsinki.

### 2.2. Study Participants

One hundred thirty-two adult smokers were enrolled in this trial. Inclusion criteria were ages 18 to 65 years, smoker of more than 20 cigarettes per day, a smoking history exceeding one year, and no concurrent use of prescription medication or antioxidant vitamins, or use of these in the previous three months. All study participants were interviewed and asked to complete a demographic and health information questionnaire. Study participants were randomly assigned to a 118 mL placebo (*n* = 26), 29.5 mL noni juice (*n* = 51), or 118 mL noni juice dose group (*n* = 55). Males and females were enrolled in equal proportions.

### 2.3. Noni Fruit Juice and Placebo

The European Union-approved form of noni fruit juice (Tahitian Noni Juice, Morinda Inc., Provo, Utah) was used for this trial. The placebo consisted of a blend of grape and blueberry juices and natural cheese flavor to mimic the flavor of the noni juice. It also served as an iridoid deficient fruit juice control.

### 2.4. Intervention

Participants and investigators were blinded to treatment group assignments. Those in the 29.5 mL group were asked to drink the noni juice all at once in the morning and on an empty stomach. Those in the other two groups were asked to drink 59 mL twice daily (118 mL daily total), once in the morning on an empty stomach and once before bedtime. This schedule was followed for 30 days. Participants were not asked to alter their smoking habits during the intervention period, and an assumption was made that they continued to smoke in the same manner (amount and duration) as they had before enrollment in the trial.

### 2.5. Serum Preparation and Analysis

Ten mL of whole blood was drawn from each participant upon enrollment and again at completion of the intervention period. Blood samples were drawn into tubes and held at 4°C for 2-3 hours, then centrifuged at 1,500 ×g for 20 minutes to separate the serum. Serum samples were sent to the Central Laboratory of the University of Illinois Chicago Medical Center for lipids, hs-CRP, and homocysteine analyses.

### 2.6. Statistical Analysis and Data Interpretation

A power analysis was performed to estimate the number of cases needed to detect a significant effect [34]. Since the study was designed to compare pre- and postdata, all analyses were conducted on paired cases in each group. To assess the influence of noni juice and placebo on the serum measurements, averages were compared before and after the intervention in each group, as well as between noni juice and placebo groups, using a paired Student's *t*-test.

### 2.7. Chemical Analyses

Iridoid contents, specifically deacetylasperulosidic acid (DAA) and asperulosidic acid (AA), were determined by high performance liquid chromatography (HPLC), according to a previously reported method [35]. HPLC grade acetonitrile (MeCN), methanol (MeOH), and water were obtained from Sigma-Aldrich (St. Louis, MO, USA). Analytical grade formic acid was purchased from Spectrum Chemical Mfg. Corp. (New Brunswick, NJ, USA). Deacetylasperulosidic acid (DAA) and asperulosidic acid (AA) standards were isolated from authentic noni fruit in our laboratory. Their identification and purities were determined by HPLC, mass spectrometry, and nuclear magnetic resonance (NMR) to be higher than 99%. They were accurately weighed and then dissolved in an appropriate volume of MeOH to produce corresponding stock solutions. The working standard solutions of DAA and AA for the calibration curves were prepared by diluting stock solutions with MeOH in seven concentration increments ranging from 0.00174–1.74 and 0.0016–0.80 mg/mL, respectively. All stock and working solutions were maintained at 0°C. Samples of noni juice and placebo were diluted with MeOH-H_2_O (1 : 1) and then filtered through a 0.45 *μ*m nylon membrane filter. Chromatographic separation was performed on a Waters 2690 Separations Module coupled with a 996 Photodiode Array (PDA) detector, equipped with a C18 column (4.6 mm × 250 mm; 5 *μ*m, Waters Corporation, Milford, MA, USA). The pump was connected to two mobile phases, A; MeCN, and B; 0.1% formic acid in H_2_O (v/v), and eluted at a flow rate of 0.8 mL/min. The mobile phase was programmed consecutively in linear gradients as follows: 0–5 min, 0% A; 40 min, 30% A. The PDA detector was monitored in the range of 210–400 nm. The injection volume was 10 *μ*L for each of the sample solutions. The column temperature was maintained at 25°C. Data collection and integration were performed using Waters Millennium software version 32.

Analyses of other major secondary metabolites in noni fruit were also performed by HPLC, according to a previously reported method [36]. Scopoletin, rutin, quercetin, and chlorogenic acid standards were accurately weighed and then dissolved in an appropriate volume of MeOH/MeCN to produce corresponding stock and working standard solutions. Chromatographic separation was performed on a Waters 2690 Separations Module coupled with a 996 PDA detector and equipped with a C18 column. The mobile phase system was composed of three solvents: A; MeCN, B; MeOH, and C; 0.1% TFA in H_2_O (v/v). The mobile phase was programmed consecutively in linear gradients as follows: 0 min, 10% A, 10% B, and 80% C; 15 min, 20% A, 20% B, and 60% C; 26 min, 40% A, 40% B, and 20% C; 28–39 min, 50% A, 50% B, and 0% C; 40–45 min, 10% A, 10% B, and 80% C. The elution was run at a flow rate of 1.0 mL/min. The UV spectra were quantified at 365 nm.

Total polyphenols were determined by the Folin-Ciocalteu method. Samples were centrifuged and diluted 1 : 10 with deionized water. The diluted samples (10 *μ*L) were mixed with 800 *μ*L deionized water and 50 *μ*L Folin-Ciocalteu (2 N). Following incubation at room temperature for a few minutes, 150 *μ*L NaCO_3_ (saturated) was added, sample tubes shaken and allowed to incubate at room temperature for 2 hours. Vehicle blanks and gallic acid standards were prepared in the same manner. Following incubation, the absorbance of the blanks, standards, and samples was measured at 765 nm in a microplate reader. Absorbance versus gallic acid concentration was used to create a calibration curve. This curve was used to determine the total phenol content of the samples. As noni fruit is a source of vitamin C [37], concentrations of this vitamin in both the placebo and noni juice product were also measured after pasteurization and bottling.

## 3. Results

### 3.1. Participant Demographics

There were no significant differences in demographics between the groups. A 1 : 1 gender ratio was maintained in each group, with mean ages ranging from 37 to 43 yr. The average number of cigarettes smoked per day in each group was from 26 to 28.6, and average pack-years (number of cigarettes smoked daily multiplied by years of smoking) ranged from 32.12 to 32.49. The ethnic compositions of each group were primarily Caucasian, 76–95%, and African American, 8–22%. Finally, the 118 mL noni juice group had a larger proportion of missed doses than the 29.5 mL group. The proportions of individuals missing <5 doses in the 29.5 mL and 118 mL noni juice groups were 30% and 32%, respectively. The rates of those missing 5 to 10 doses in the same low- and high-dose groups were 2% and 10%, respectively.

### 3.2. Changes in Lipid Profiles, hs-CRP, and Homocysteine

Total cholesterol, triglycerides, and hs-CRP of the placebo and noni juice groups are compared in Table 1. No significant changes were observed in the placebo group during the trial, even though there was a slight trend of increased values after the 30-day period. In both noni juice groups, decreases in mean cholesterol, triglyceride, and hs-CRP were observed. Posttrial values were significantly lower than pretrial and placebo group values. The sole exception was posttest hs-CRP in the 29.5 mL noni juice group, where it was significantly lower than a mean posttest hs-CRP of the placebo group (*P* < 0.05) but not significantly different from the baseline average (*P* > 0.05). In the noni juice groups, average total cholesterol, triglycerides, and hs-CRP decreased by 20.3–25.6%, 29.4–41.2%, and 15.2%, respectively. A comparison of posttrial values of both noni juice groups revealed no significant differences. This may indicate a possible threshold of antioxidant activity that is reached by a daily dose of 29.5 mL but may also be due to lower compliance in the 118 mL noni group.

A stratified analysis of the aggregate baseline and posttest total cholesterol, LDL, and triglyceride results of both noni juice groups is provided in Table 2. In this analysis, stratification is based on ranges of baseline, or initial, values of total cholesterol or triglycerides. Low, middle, and high strata for baseline serum cholesterol levels were 190–219 mg/dL, 220–299 mg/dL, and >300 mg/dL, respectively. Low, middle, and high strata for baseline triglyceride levels were 170–199 mg/dL, 200–399 mg/dL, and >400 mg/dL, respectively. The purpose in such an analysis is to evaluate the effect of noni juice relative to the degree of deviation from normal population values. The decreases in total cholesterol, LDL, and triglycerides that occurred in the lowest strata of ranges were 6.6, 8.9, and 9.5%, respectively. Decreases in the same measurements in the middle strata were 18.1, 22.9, and 21.6%, respectively. Corresponding trends in the high strata of ranges were 22.0, 28.2, and 53.8%, respectively, but only the total cholesterol change reached statistical significance. This stratified analysis reveals that the magnitude of effect increased as initial cholesterol or triglycerides levels increased.

Stratified analyses of total cholesterol and triglycerides by noni juice dose are provided in Table 3. Significant declines in serum total cholesterol and triglycerides were observed in the middle strata of each dose group. In the 29.5 mL noni juice group, significant decreases occurred in cholesterol of the low stratum as well as in triglycerides of the high stratum. A trend of greater reductions within higher baseline strata is also apparent. Decreases in mean total cholesterol in the low, middle, and high baseline strata of the 29.5 mL group were 12.1, 17.3, and 36.4%, respectively. In the low, middle, and high strata of this dose group, triglycerides declined by 10.5, 29.3, and 61.8%, respectively. Decreases in total cholesterol in the low, middle, and high strata of the 118 mL group were 9.9, 29.2, and 23.6%, respectively, with corresponding declines of 12.6, 25.2, and 41.7% in serum triglycerides. Within each dose group, greater declines in mean values were associated with greater initial cholesterol or triglycerides levels. The only exception was for the percent decline in total cholesterol in the 118 mL group, where the mid-stratum decrease was greater than that of the high stratum.

Homocysteine levels were reduced in the noni juice groups. The aggregate mean (±standard deviation) at baseline (19.7 ± 8.5 *μ*mol/L) declined by 23.9% to 15.0 ± 9.0 (*P* < 0.05). Conversely, aggregate mean HDL in the noni juice groups increased from 49 ± 10 to 57 ± 9 mg/dL (*P* < 0.05).

### 3.3. Adverse Events

No adverse events were observed in the placebo or noni juice groups during the intervention period.

### 3.4. Chemical Comparison of Noni Juice and Placebo

The phytochemical compositions of the noni juice product and placebo used in this trial are compared in Table 4. The total polyphenol content of each of these was similar, with no substantial difference in flavonoid (quercetin and rutin) or chlorogenic acid concentrations. The vitamin C contents of noni juice and placebo were not significant, with both being less than 0.2 mg/mL. Iridoids, which were present in significant quantities in the noni juice product, were absent in the placebo. Scopoletin was also detected in noni juice, but the content was minor compared to the total iridoid concentration and was below the quantity previously demonstrated to provide effective antioxidant action [38, 39].

## 4. Discussion

Serum total cholesterol, LDL, triglycerides, hs-CRP, and homocysteine were lowered in heavy smokers within 4 weeks of noni juice ingestion. This observation is consistent with previous reports in which supplementation with antioxidant nutrients and plant extracts inhibited cigarette smoke-induced dyslipidemia *in vivo* and in human intervention studies [40–43]. It is also important to note that noni juice did not produce any changes in human trials where participants were already within normal healthy ranges. For example, four weeks of daily supplementation of 30, 300, or 750 mL noni juice did not cause any change in total cholesterol, LDL, HDL, or triglycerides in 96 healthy volunteers with normal lipid profiles and who were light smokers (<5 cigarettes/day) or nonsmokers [44]. Also, no changes occurred in total cholesterol, LDL, HDL, or triglycerides levels in 34 diabetic patients, each with existing normal lipid profiles, after consuming 30 mL noni juice/day for 21 days [45]. Considering this, it is likely that the decline in elevated blood lipids observed in our study was due to the inhibition of oxidative stress by noni juice.

The blood lipid profiles of heavy smokers were improved after 4 weeks of noni juice ingestion, even when compared to a fruit juice placebo. The phytochemical analysis of the noni juice product and the placebo indicates that iridoids, specifically deacetylasperulosidic acid and asperulosidic acid, are the major point of difference. Iridoids are known for antioxidant activities [46]. Oleuropein, a secoiridoid, is perhaps the best characterized, relative to its antioxidant capacity [47–49]. Similar to the results in our clinical trial, oleuropein lowered total cholesterol, triglycerides, and LDL while increasing HDL *in vivo*. These effects were associated with increases in antioxidant enzyme activities [50]. Further, olive leaf extract, a rich source of oleuropein, was found to reduce total cholesterol in a human intervention study [51].

Data demonstrating the antioxidant potential of iridoid glycosides are also emerging. Two iridoids that are structurally similar to those found in noni fruit are loganic acid and loganin. Loganic acid reduced superoxide generation in human neutrophils activated by N-formyl-methionylleucyl-phenylalanine and arachidonic acid in a concentration-dependent manner [52]. Loganin exhibited antioxidant activities in rat renal mesangial cell cultures incubated in the presence of advanced glycation end products, inducers of cellular oxidative stress. Cells incubated with loganin for 48 hours exhibited increased antioxidant enzyme activity, such as superoxide dismutase and glutathione peroxidase activities, and decreased malondialdehyde concentration [53].

Similar to the antioxidant activity of oleuropein in olive, iridoids occurring in noni fruits inhibited the oxidation of low-density lipoproteins (LDL) *in vitro*. Deacetylasperulosidic acid and asperulosidic acid both demonstrated significant inhibition of copper sulfate-induced oxidation of human LDL [54]. Also, deacetylasperulosidic acid and asperulosidic acid prevented 4-nitroquinoline 1-oxide- (4NQO-) induced DNA damage *in vitro* [37]. 4NQO is a genotoxin that causes the formation of 8-hydroxydeoxyguanosine (8OHdG), a product of DNA oxidation. 4NQO exposure leads to the formation of superoxide, hydrogen peroxide, and hydroxyl radicals, resulting in the production of a substantial amount of 8OHdG in DNA in mammalian and bacterial cells [55, 56]. Treatment with deacetylasperulosidic acid and asperulosidic acid reduced 4NQO genotoxicity in prokaryotic cells by 98.96 and 99.22%, respectively. Therefore, the *in vitro* oxidative activity of 4NQO was almost entirely abolished by the addition of either iridoid.

The antioxidant activity of iridoids in noni and the lack of iridoids in the placebo suggest that they are responsible for at least some of the protective or adaptive effects of noni juice observed in this trial. Several human studies have revealed that cigarette smoke reduces glutathione peroxidase and glutathione transferase activities [57–62]. It is interesting to note that noni juice produced a larger decrease in plasma lipid hydroperoxide than plasma superoxide anion radicals in a previous human trial [33], indicating that noni juice may be effective in increasing the activities of glutathione peroxidase and glutathione transferase. This possibility is further supported by the observation that the activities of these enzymes were doubled in streptozotocin-induced diabetic rats fed an ethanolic extract of noni fruit for 30 days [63]. Therefore, it is possible that noni juice inhibits cigarette smoke-induced oxidative stress, and subsequent dyslipidemia, by increasing the activity of glutathione utilizing enzymes.

## Figures and Tables

**Table 1:** Comparison of baseline and posttest serum measurements among placebo and noni juice groups.

Dose group and sampling time	Total cholesterol (mg/dL)	Triglycerides (mg/dL)	hs-CRP (mg/L)
Placebo			
baseline	239.2 ± 9.4	254.2 ± 54.2	1.48 ± 2.16
posttest	246.6 ± 16.9	281.7 ± 37.3	2.22 ± 1.79
29.5 mL noni juice			
baseline	250.2 ± 36.75	325.9 ± 152.0	1.61 ± 1.55
posttest	186.2 ± 53.3^a^	191.5 ± 131.4^a^	1.39 ± 1.36^b^
118 mL noni juice			
baseline	242.3 ± 43.5	310.4 ± 163.4	1.72 ± 1.51
posttest	193.2 ± 70.5^a^	218.8 ± 150.8^b, c^	1.46 ± 1.39^b, c^

^a^ *P* < 0.001 compared to placebo (posttest) and baseline, ^b^ *P* < 0.05 compared to placebo (posttest), and ^c^ *P* < 0.05 compared to baseline.

**Table 2:** Total cholesterol (TC), LDL, and triglyceride levels within specified baseline ranges among the aggregate noni juice group (both doses combined).

Baseline range	Baseline	Posttest
Total cholesterol (TC)		
190–219 mg/dL	204.4 ± 9.3	190.8 ± 41.6
220–299 mg/dL	249.1 ± 21.5	203.9 ± 69.4^a^
>300 mg/dL	328.7 ± 22.8	256.4 ± 89.3^b^
LDL		
TC 190–219 mg/dL	154.6 ± 10.3	140.8 ± 21.6^b^
TC 220–299 mg/dL	199.1 ± 20.5	153.4 ± 1.4^a^
TC > 300 mg/dL	287.7 ± 20.8	206.4 ± 39.3
Triglycerides (Trig)		
170–199 mg/dL	184.1 ± 7.5	166.6 ± 95.3^b^
200–399 mg/dL	266.7 ± 48.0	209.1 ± 129.3^b^
>400 mg/dL	553.6 ± 143.5	255.5 ± 219.7

^a^ *P* < 0.001, ^b^ *P* < 0.05 compared to baseline.

**Table 3:** Total cholesterol (TC) and triglyceride levels within specified baseline ranges among noni juice dose groups.

Baseline range	29.5 mL Noni juice		118 mL Noni juice	
	Baseline	Posttest	Baseline	Posttest
Total cholesterol (TC)				
190–219 mg/dL	200.1 ± 7.1	175.8 ± 50.0^a^	206.4 ± 10.5	186.0 ± 59.7
220–299 mg/dL	236.2 ± 16.8	195.3 ± 50.9^a^	262.8 ± 17.9	186.1 ± 74.1^a^
>300 mg/dL	323.7 ± 18.6	205.7 ± 84.3	328.8 ± 27.8	251.2 ± 87.3
Triglyceride (Trig)				
170–199 mg/dL	184.6 ± 6.1	165.2 ± 136.4	185.5 ± 7.0	162.1 ± 58.6
200–399 mg/dL	273.5 ± 52.2	193.3 ± 141.5^a^	267.9 ± 8.8	200.4 ± 29.2^a^
>400 mg/dL	547.4 ± 111.3	208.9 ± 109.0^a^	583.6 ± 187.8	340.3 ± 311.7

^a^ *P* < 0.05 compared to baseline.

**Table 4:** Phytochemical compositions (mean ± standard deviation) of the noni juice product and placebo evaluated in the clinical trial.

Analysis	Noni juice	Placebo
Chlorogenic acid (mg/mL)	0.0831 ± 0.0015	0.1030 ± 0.0020
Rutin (mg/mL)	0.0349 ± 0.0004	0.0252 ± 0.0004
Total polyphenols (mg/mL)	0.6200 ± 0.0400	0.4900 ± 0.0600
Total iridoids (mg/mL)	0.5115 ± 0.0162	none detected
Deacetylasperulosidic acid (mg/mL)	0.3747 ± 0.0158	none detected
Asperulosidic acid (mg/mL)	0.1122 ± 0.0070	none detected
Scopoletin (mg/mL)	0.0139 ± 0.0005	none detected
